# Supplementary material for: Cloud BioLinux: pre-configured and on-demand bioinformatics computing for the genomics community
Source: BMC Bioinformatics. 2012 Mar 19;13:42. doi: 10.1186/1471-2105-13-42 (PMC3372431; doi:10.1186/1471-2105-13-42)
Supplement: Additional file 1 — Supplementary 1 Cloud BioLinux software documentation in the form of a mini, self-contained website. Users need to download and uncompress the .zip file, and open through a web browser the "index.html" file available on the main directory. (ZIP 1823 kb). [file 1471-2105-13-42-S1.ZIP › Cloud-BioLinux-Package-Documentation/docs/act.html]

Bio-Linux Software Documentation Pages

Back to search form

## act

|  |  |
| --- | --- |
| Name | act |
| Description | **ACT** (Artemis Comparison Tool) is a DNA sequence comparison viewer based on Artemis. In common with Artemis, ACT is written in Java and runs on UNIX, GNU/Linux, Macintosh and MS Windows systems. It can read complete EMBL and GENBANK entries or sequence in FASTA or raw format. Extra sequence features can be in EMBL, GENBANK or GFF format. ACT is distributed under the same license as Artemis.  The sequence comparison displayed by ACT is usually the result of a blastn or tblastx search that has been processed by MSPcrunch. MSPcrunch must be run with the -d flag for the output to be usable by ACT.  ACT is also available online in the form of WebACT. This provides a database of sequence comparisons between all publicly available prokaryotic genome sequences, allowing the on-line visualisation of comparisons between up to five genomic sequences. WebACT can be found here |
| Homepage | http://www.sanger.ac.uk/Software/ACT/ |
| Remote Documentation | http://www.sanger.ac.uk/Software/ACT/manual/ |

|  |
| --- |
| Please see Remote Documentation |
